# Mechanistic Role of the Mdm2/MdmX Lid Domain in Regulating Their Interactions with p53

**DOI:** 10.3390/biom15050642

**Published:** 2025-04-30

**Authors:** Qiuyin Wei, Chenqi Li, Yibing Tang, Jinping Bai, Wang Li, Jidong Liu, Zhengding Su, Xiyao Cheng

**Affiliations:** 1 Institute of Modern Fermentation Engineering and Future Foods, School of Light Industry and Food Engineering, Guangxi University, No. 100, Daxuedong Road, Nanning 530004, China; 2216392016@st.gxu.edu.cn (Q.W.); 2416393005@st.gxu.edu.cn (C.L.); 2405120101@st.gxu.edu.cn (Y.T.); 2316392001@st.gxu.edu.cn (J.B.); 2416393006@st.gxu.edu.cn (W.L.); liujd@gxu.edu.cn (J.L.); 2 School of Pharmaceutical Sciences and Institute of Materia Medica, Xinjiang University, Urumqi 830017, China; james_su@xju.edu.cn

**Keywords:** tumor suppressor protein p53, Mdm2, MdmX, Lid

## Abstract

p53 functions as a critical guardian of the genome, orchestrating tumor suppression pathways and ensuring the integrity of chromosomal stability. Mdm2 and MdmX, homologous proteins, serve as negative feedback regulators of p53. In approximately half of tumor cases, overexpression of Mdm2/MdmX results in the inhibition of p53 activity. Current research focuses on designing Mdm2 and MdmX inhibitors based on the structure of lidless N-terminal forms of these proteins. However, growing evidence suggests that the lid of Mdm2 and MdmX plays a key role in the selective binding of p53 and inhibitors. Therefore, targeting the lid in the screening and design of Mdm2/MdmX inhibitors may offer a novel strategy for developing anti-cancer drugs. This review examines the impact of the Mdm2/MdmX lid on ligand binding, providing valuable insights for future research and guiding new approaches to the screening and design of innovative anti-cancer therapeutics.

## 1. Introduction

The World Health Organization has identified cancer as the second leading cause of death worldwide, with effective tumor treatment continuing to pose a significant challenge in the medical field. Research has shown that the tumor suppressor protein p53 plays a critical role in inhibiting tumor growth and maintaining genetic stability [1].

In approximately 50% of tumor cases [2,3], wild-type p53 (WTp53) is often suppressed, mainly due to the overexpression of its negative regulators, mouse double minute 2 (Mdm2) and mouse double minute X (MdmX), which mediate the negative feedback regulation of p53 [4]. MdmX and Mdm2 inhibit p53’s transcriptional activity by binding to its transactivation domain (TAD) [5]. Additionally, Mdm2, possessing E3 ubiquitin ligase activity, promotes the ubiquitin-proteasomal degradation of p53 upon binding to its N-terminal TAD [6,7]. Furthermore, MdmX can enhance p53’s ubiquitination and degradation through its interaction with Mdm2 [5,8]. As a result, inhibiting the activity of Mdm2 and MdmX to reactivate WTp53 is considered a promising strategy for developing anti-cancer therapies.

**Figure 1:**
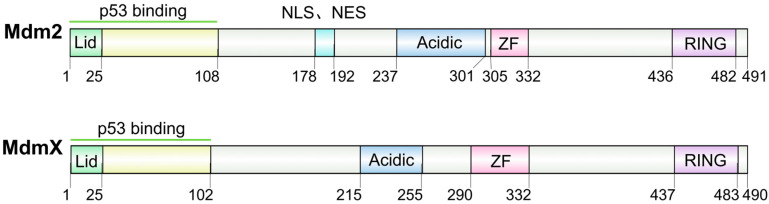
Schematic representation of the Mdm2 and MdmX structures (Image generated using IBS 2.0 [13]).

As shown in Figure 1, Mdm2 consists of several structural domains [4,9]: an N-terminal p53-binding domain that interacts with the N-terminal transactivation domain (TAD) of p53; a nuclear localization signal (NLS); a nuclear export signal (NES); an acidic domain (Acidic) in the central region; a zinc finger (ZF) domain; and a C-terminal RING finger (RING) domain. MdmX shares high structural similarity with Mdm2 but, unlike Mdm2, lacks both the NLS and NES [4,10,11]. The p53-binding domain of Mdm2/MdmX spans approximately 105 amino acids, with the 25 N-terminal amino acids forming an intrinsically disordered region (IDR). This IDR has been shown to act as a pseudo-substrate motif that occludes the p53-binding site, thus it is referred to as the lid [12].

Current research on Mdm2/MdmX primarily focuses on the mechanisms of their interaction with p53, the regulation of this interaction, and the development of Mdm2 and MdmX inhibitors that activate p53’s tumor-suppressing activity [14,15,16]. Early Mdm2 inhibitors showed minimal differences in affinity between full-length Mdm2 and truncated Mdm2 (typically residues 17–125, lacking the lid) [17,18], which led researchers to remove the disordered intrinsically disordered region (IDR) lid to stabilize the p53-binding domain and better understand its binding mechanism with ligands. However, in late 2012, Michelsen et al. [19] reported a significant disorder-to-order transition in the lid region of Mdm2 upon 00binding to the ligand Pip2. This discovery shifted the focus to the functional role of the Mdm2/MdmX lid. Although extensive research has been dedicated to the lid of Mdm2, investigations into the N-terminal lid of MdmX remain limited, and the precise functional role of MdmX has yet to be fully characterized. Since Mdm2 and MdmX are homologous proteins with highly similar p53-binding domains, we hypothesize that the lid of MdmX may serve a comparable function. During the p53 binding process, the lids of Mdm2 and MdmX may compete for the binding pocket with p53 [20], with different modifications of the lid playing distinct regulatory roles [21]. Exploring these mechanisms could open new avenues for designing effective MdmX-targeted inhibitors. Furthermore, the p53-Mdm2/MdmX interaction may provide a valuable model for understanding protein–protein interactions, particularly for proteins with intrinsically disordered regions.

The high flexibility of the lid region in Mdm2/MdmX proteins presents significant challenges in capturing its electron density via X-ray diffraction, thereby complicating structural and functional investigations of the lid. Current research on the Mdm2/MdmX lid primarily utilizes techniques such as molecular dynamics (MD) simulations, nuclear magnetic resonance (NMR) spectroscopy, isothermal titration calorimetry (ITC), surface plasmon resonance (SPR), and fluorescence polarization (FP). These studies focus on the dynamic conformational changes in the lid, its impact on Mdm2/MdmX binding to p53 and other small-molecule ligands, and the role of lid phosphorylation in modulating the binding of Mdm2/MdmX to p53 and other ligands. This review summarizes the current research landscape in these areas, aiming to provide valuable insights for further studies on the Mdm2/MdmX lid and to offer a theoretical basis for developing effective Mdm2/MdmX inhibitors.

## 2. Conformational Behavior of the Lid in the Free (Apo) and p53-Bound (Holo) States

### 2.1. Detection of the Conformational Dynamics of the Lid Using NMR Method

Protein conformational changes involve the movement of amino acid side chains and domains, occurring on time scales from picoseconds to seconds. During this dynamic process, the protein passes through various transition states, which are essential for understanding its function and for drug design. NMR is a powerful tool for studying dynamic protein structures, and due to the intrinsically disordered nature of the lid, it is particularly well-suited for investigating its conformation.

**Figure 2:**
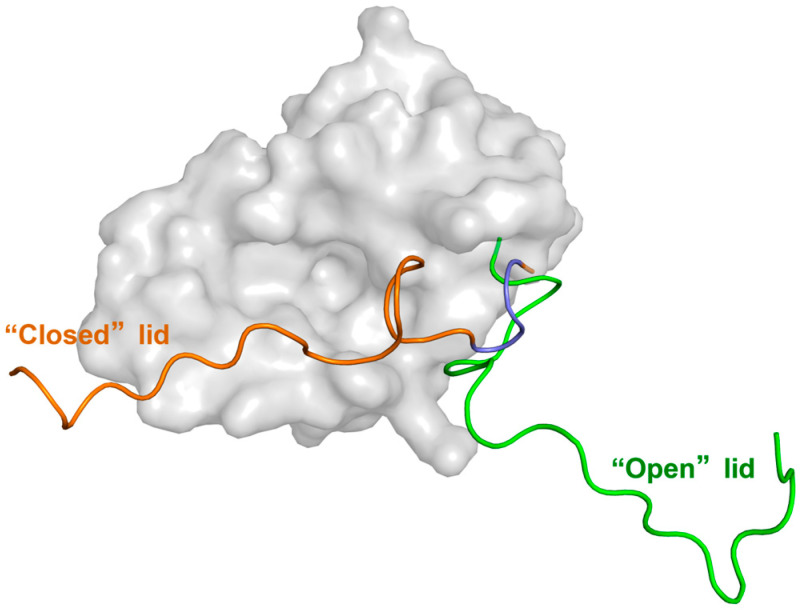
Conformational states of the Mdm2’s lid. The green structure corresponds to the ‘open’ state lid, while the orange structure represents the ‘closed’ state lid. The latter contains residues 21–24, which exhibit a distinct α-helix propensity, highlighted in Slate (PDB ID: 1Z1M [24]).

In 2003, McCoy et al. [12] conducted an NMR study on Mdm2^16–109^ and found that, in addition to the ordered residues 25–109, residues 16–24 form a lid. Part of this lid interacts with Mdm2’s p53-binding site (referred to as the Mdm2 binding pocket), suggesting that the lid may help stabilize Mdm2’s conformation and weakly compete with both peptide and non-peptide inhibitors. Building on this, Showalter et al. [22] performed an NMR dynamics study on Mdm2^17–125^ and proposed that, in the apo state, the lid exists in a dynamic equilibrium between a “closed” conformation (major, 90%) and an “open” conformation (minor, 10%), with a slow conversion rate. The conformational behavior of the lid is shown in Figure 2. In the “closed” state, the lid adopts an ordered conformation, with residues 21–24 (Ala-Ser-Glu-Gln) exhibiting a pronounced α-helical tendency, blocking the Mdm2 binding pocket. In the “open” state, the lid adopts a disordered, high-entropy form [22,23,24], moving away from the binding pocket and allowing ligands to enter. Further studies by Watanabe et al. [25] revealed that truncating residues 1–16 from the lid increases the proportion of Mdm2 in the “open” state. Additionally, increased temperature favors the “open” state, and the chemical shift changes in Ile19 and Met50 in truncated Mdm2^16–109^ show a trend similar to that observed with rising temperature. These findings suggest that residues 1–16 of the lid play a role in stabilizing the “closed” state. Moreover, NMR studies by Zhan et al. [26] demonstrated that the p53 peptide (p53p) can compete with the lid for binding to Mdm2, leading to the lid adopting a fully disordered state. This strongly supports the idea that the lid obstructs the p53-binding site in apo-Mdm2, keeping the lid in the “closed” state.

### 2.2. Investigating the Function of the Lid Through MD Simulation

MD simulation is a vital tool for analyzing protein structures and investigating protein functions. Numerous research teams have used MD simulations to study the conformational changes in the protein lid and how the lid region affects the p53-Mdm2 interaction.

**Figure 3:**
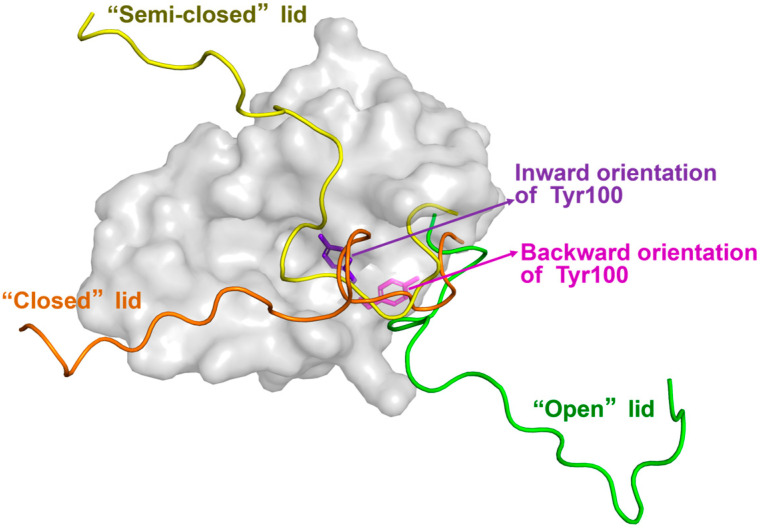
Different orientations of Tyr100 in the various conformational states of the Mdm2 lid. In the ‘closed’ state (orange), the side chain of Tyr100 (purple) points inward, toward the Mdm2 binding pocket. In the ‘open’ state (green), the side chain of Tyr100 (magenta) is oriented away from the Mdm2 binding pocket (PDB ID: 1Z1M [28]). The yellow structure represents the ‘semi-closed’ state of the lid.

The simulation results of Pantelopulos et al. [27] suggest that Mdm2 must transition from the apo “closed” state to the “open” state in order to bind p53. In the apo state, Mdm2 exhibits a certain degree of flexibility, allowing an easy shift between the apo-like and holo-like conformations. Mukherjee et al. [28] developed a Markov State Model (MSM) from extensive unbiased simulation trajectories of apo-Mdm2. Their findings revealed that in the holo state, the Mdm2 lid is fully displaced by p53 and adopts an “open” conformation. Additionally, the “closed” and “open” states undergo slow (>10 ms) two-state exchange. Dastidar et al. [29,30,31] utilized MD simulations to investigate interactions between the lid and residues near the Mdm2 binding pocket. They first observed that the lid affects the orientation of Tyr100’s side chain. In the “closed” state, Tyr100’s side chain faces inward, blocking p53p binding. In the “open” state, Tyr100 rotates away from the binding pocket, exposing it and facilitating p53p binding [29,30]. These two conformations of Tyr100 are shown in Figure 3. Further analysis revealed that the negatively charged residues Asp11 and Glu23 on the lid form salt bridges with the positively charged residues Lys94 and Arg97 in the Mdm2 binding pocket. Additionally, Ile19 interacts hydrophobically with Leu54, His96, and Tyr100 in the binding pocket, stabilizing the lid in the “closed” conformation [31]. Furthermore, an intermediate conformation between the “closed” and “open” states was identified. In this state, hydrogen bonds between Asn3, Asn5, and Lys94, as well as between Asp11, Tyr67, and Gln72, disrupt the salt bridge between Asp11 and Lys94, causing the lid to partially detach from the Mdm2 pocket [31]. Verkhivker [32] and Bueren-Calabuig [33] referred to this intermediate conformation as the “semi-closed” state, as shown in Figure 3. The lid fluctuates between the “closed,” “semi-closed,” and “open” states. In the “semi-closed” state, the lid blocks p53 binding while allowing smaller molecules to enter the Mdm2 pocket.

The MD simulation study of Ciemny et al. [34] suggests that in the “closed” state, the lid residues 21–24 of Mdm2 may adopt a p53p-like helical structure, exhibiting a certain degree of stability. The study also found that the lid interacts hydrophobically with core residues in the Mdm2 binding pocket, particularly through Leu27 and Ile19. Additionally, the frequency of contact between the lid and the Mdm2 binding pocket was found to be comparable to that of p53p with the Mdm2 pocket, suggesting that p53 competes with the lid for binding to Mdm2. In a recent study, Watanabe et al. [25] utilized gREST-enhanced sampling techniques to more flexibly delineate the “solute” (target) and “solvent” (surrounding) regions, facilitating a more precise characterization of lid conformations. They found that the apo-Mdm2 “closed” state exists as two distinct conformations that rapidly interconvert. Furthermore, they discovered that the lid must pass through a “semi-closed” state before transitioning to the “open” state. In this “semi-closed” conformation, the first 14 residues of the lid are displaced from the Mdm2 binding pocket, while residues 15–18 retain a helical structure similar to that of the “closed” state.

Regarding the lid’s states, Mendoza-Martinez et al. [35], through MD simulations, introduced a new perspective: the conformation of the apo-Mdm2 lid (residues 1–16) can be classified into two main states: the “closed and disordered” state and the “open and ordered” state. These two conformations account for 75–80% and 6–8% of the conformational ensemble, respectively. Notably, the “open and disordered” state, previously mentioned, represents less than 1% in this simulation, due to its unstable energy.

### 2.3. The Regulatory Mechanism of the Lid Investigated Through Affinity Assays

Zhan et al. [26] determined the K_d_ values of Mdm2^1–109^ with the lid and Mdm2^25–109^ without the lid, binding to various lengths of p53p (p53p^19–26^, p53p^17–28^, p53p^15–29^) using SPR. The results are shown in Table 1.

The comparison shows that the affinity of Mdm2^1–109^ with the lid for p53p is lower than that of Mdm2^25–109^ without the lid, with the affinity increasing as the length of p53p increases. This suggests that the presence of the lid in the Mdm2-ligand binding process introduces steric hindrance, weakening binding in a ligand size-dependent manner. In a study by Chen et al. [21] using FP and SPR to examine the interaction between MdmX and p53p, they found that the lid reduced the affinity by 1.7 to 2.8 times. Compared to the Mdm2 lid, the MdmX lid only slightly weakened the binding of the peptide to MdmX, exerting a lesser effect on p53p binding.

Summary of the key points from the above content: The lids of Mdm2 undergo dynamic equilibrium between the ‘closed’ (predominant) and ‘open’ (minor) conformations, with the ‘open’ state facilitating the binding of p53. Several important insights into the lid’s influence on Mdm2-p53 binding have emerged: First, residues 21–24 of the Mdm2 lid may form a slightly stable p53p-like helical structure, which, through interactions with surrounding residues in the Mdm2 binding pocket, occludes the site. In the “closed” state, the lid blocks p53 binding. Second, for Mdm2 to bind p53, the lid must transition from the “closed” to the “open” state, thereby exposing the binding pocket to accommodate p53. Additionally, the lid regulates the size of the Mdm2 pocket by modulating the orientation of the Tyr100 side chain, which in turn affects p53 binding. Notably, NMR and MD simulations each have distinct strengths and limitations: NMR, as an experimental technique, provides atomic-level structural insights and focuses on the dynamic changes in the lid’s structure, while MD simulations, as a computational method, focus on molecular interactions and mechanisms over fixed time periods. Thus, the choice of research methods and analytical approaches for Mdm2/MdmX lids of varying lengths and ligands may lead to differing conclusions. This is especially relevant in MD simulations, where the selection of force field and the system’s equilibrium state can significantly influence the results. Additionally, protein affinity measurements are critical for quantifying the binding strength between ligands and proteins. In lid research, these assays provide valuable insights, offering a more direct reflection of the lid’s impact on Mdm2/MdmX-ligand binding. To mitigate the limitations of individual techniques, the integration of methods such as NMR, MD simulations, and affinity assays provides a comprehensive and robust understanding of the structural function of the lid and its regulatory mechanisms in Mdm2/MdmX.

## 3. The Influence of Protein Phosphorylation Modifications on the Binding of Mdm2/MdmX to p53

### 3.1. The Impact of Lid Ser17 Phosphorylation on Mdm2-p53 Binding

Under stress conditions, the lid residue Ser17 of Mdm2 is highly prone to phosphorylation [36]. McCoy et al. [12], through NMR studies of Mdm2^16–125^, discovered that simultaneous phosphorylation of Ser17 on the lid and Thr18 [37] and Ser20 [38] on p53p^16–24^ impedes the binding of p53 to Mdm2 [39]. Since then, a large number of research teams have turned their focus to investigating the effects of Ser17 phosphorylation on the lid and its impact on the structure and function of the Mdm2 protein.

In a recent study, Watanabe et al. [25] utilized ITC to evaluate the affinity between p53p^15–29^ and Mdm2 with the lid, as well as Mdm2^S17D^ (where Ser17 is mutated to Asp, mimicking the negatively charged phosphate group after phosphorylation) and Mdm2^S17K^ (where Ser17 is mutated to Lys, introducing a positively charged group), both incorporating the lid. Compared to Mdm2, the affinity between Mdm2^S17D^ and p53p^15–29^ was reduced by a factor of four, while Mdm2^S17K^ increased the affinity by two-fold, suggesting that Ser17 phospho-mimicry disrupts the binding of Mdm2 to p53. Dastidar et al. [31], through MD simulations of Mdm2^pS17^ (the Ser17 site is phosphorylated) and Mdm2^S17D^, observed that when the phosphate group on pSer17 forms a salt bridge with Lys51 or Lys94 near the Mdm2 binding pocket, the lid adopts a relatively stable “closed” state. However, when pSer17 forms a salt bridge with Arg97 or Lys98, the lid transitions to an “open” state. The “closed” state predominates, while the “open” state occurs less frequently. In simulations of Mdm2^S17D^ with phosphor-mimicry, although Asp17 can form salt bridges with Lys51 or Lys94, in the final 2 ns of a 10 ns trajectory, Asp17 stabilizes near Lys97 and Arg98, causing the lid to favor the “open” state. MD simulations by Bueren-Calabuig et al. [23] revealed that in Mdm2^pS17^, the lid transitions only between the “closed” and “semi-closed” states, with no fully “open” conformation detected. In comparison to Mdm2^pS17^, the lid of Mdm2^S17D^ exhibits a weaker interaction with the Mdm2 binding pocket, adopting a more extended “semi-closed” conformation. Verkhivker’s MD simulations [32] on Mdm2^16–125^ with a truncated lid showed that in Mdm2^pS17^, the lid predominantly exists in the “closed” state, stabilized by salt bridges between pSer17 and Lys94, along with hydrogen bonds with His73 and Gln72. In contrast, the lid in Mdm2^S17D^ rapidly switches between the “closed” and “semi-closed” states. Molecular docking further revealed that this rapid switching is primarily due to the weaker salt bridge stability between Asp17 and Lys94. Additionally, they found that in Mdm2^pS17^, the Tyr100 side chain is oriented toward the Mdm2 binding pocket, while in Mdm2^S17D^, Tyr100 swings away from the pocket. Compared to the S17D phosphor-mimetic mutation, the interactions between the pSer17 lid and the Mdm2 binding pocket are more stable, suggesting that phosphorylation of Ser17 may hinder the formation of the p53-Mdm2 complex. From another perspective, although S17D, as a phospho-mimic, does not fully replicate the actual phosphorylation process, it provides a useful reference for understanding how phosphorylation influences molecular interactions and regulatory mechanisms.

In contrast to the previously mentioned findings, the study of Zhan et al. [26] indicates that the binding of Mdm2 to p53 is independent of phosphorylation at Ser17 in the Mdm2 lid. They observed that the HSQC spectra of the three lid-containing proteins—Mdm2, Mdm2^pS17^, and Mdm2^S17D^—were highly similar in the apo state, as determined by NMR, suggesting that phosphorylation had little effect on Mdm2’s conformation. Furthermore, affinity measurements using FP and SPR revealed no significant differences in the binding affinity between Mdm2, Mdm2^S17D^, and Mdm2^pS17^ with p53p^15–29^. Circular dichroism (CD) spectroscopy revealed that Ser17 phosphorylation only slightly increased the α-helical content of the lid and had a minor stabilizing effect on its “closed” conformation. Based on these results, the authors concluded that Ser17 phosphorylation on the Mdm2 lid does not significantly influence its interaction with p53p, which contrasts with the conclusions of previous studies.

Worrall et al. [40,41,42] proposed a third perspective using enzyme-linked immunosorbent assay (ELISA), Western blot (WB), and Differential scanning fluorimetry (DSF) experiments. They discovered that S17D phosphor-mimetic mutation enhances the stability of the Mdm2-p53 tetrameric complex, thereby promoting p53 ubiquitination. The ELISA [41] results showed that Mdm2^S17D^ exhibited significantly enhanced affinity for both the BOX-I peptide (located in the Mdm2-binding site in the N-terminal region of p53) and the BOX-V peptide (located in the DNA-binding domain of p53, which also binds Mdm2) [43,44,45]. Additional solid-phase direct protein binding assays [40,46] and DSF [42] experiments showed that the Mdm2^S17D^-p53 tetramer complex was more stable than the unmodified Mdm2-p53 complex, indicating that Mdm2^S17D^ enhances the affinity between Mdm2 and p53. Furthermore, in vitro ubiquitination assays [40,41,44] and cell experiments [41] comparing Mdm2 and Mdm2^S17D^ indicated that WB results revealed Mdm2^S17D^ increased p53 ubiquitination and reduced p53 protein levels in cells. Consistent with Worrall’s findings, intrinsic fluorescence measurements by Fraser et al. [47] demonstrated that Mdm2^S17D^ did not induce any changes in intrinsic fluorescence, suggesting that Ser17 phospho-mimicry does not necessarily promote binding between the lid and the central domain. However, their cellular experiment indicated that structural changes induced by Ser17 phospho-mimicry could regulate ubiquitin release through the E2 ubiquitin-conjugating enzyme UBCH5a, mediated by the RING domain of Mdm2. This process accelerates the E2 cycle, thereby enhancing p53 ubiquitination.

### 3.2. The Effect of Tyr55/Tyr99 Phosphorylation on the Structure and Function of the MdmX Lid

There is limited research on the structure and function of the MdmX lid, with a notable study by Chan et al. [20] utilizing MD simulations to examine how phosphorylation regulates lid function. Their results showed that phosphorylation of Tyr99 [48,49], a key amino acid near the MdmX binding pocket, causes a steric clash with Pro20 of p53p. Additionally, the phosphate group forms a strong salt bridge with Arg18 on the lid, driving the lid toward the binding pocket and shifting the conformational equilibrium toward a “closed” state, which inhibits MdmX-p53 binding. In contrast, phosphorylation of Tyr55 [49], located farther from the binding pocket thanTyr99, promotes electrostatic interactions with the lid, favoring an “open” conformation that enhances MdmX-p53 binding.

In summary, there is ongoing debate in the current research regarding whether phosphorylation of the Mdm2 lid region affects its binding to p53. Most studies suggest that phosphorylation leads to interactions between the phosphate group and core residues in the Mdm2 binding pocket, thereby occluding the pocket and preventing p53 binding. However, Zhan et al. found that phosphorylation of Ser17 on the Mdm2 lid does not significantly impact Mdm2’s ability to bind p53. Further work by Fraser et al. reveals an alternative mechanism: while Ser17 phosphorylation does not directly alter interactions between the lid and residues near the Mdm2 binding pocket, it induces conformational changes in the Mdm2 RING domain, promoting Mdm2 binding to p53 and enhancing p53 ubiquitination. Studies on MdmX phosphorylation show that phosphorylation of Tyr55 and Tyr99 in the MdmX binding pocket acts as a switch, driving conformational changes in the lid and precisely regulating MdmX’s binding to p53.

## 4. The Influence of the Lid on Mdm2/MdmX Protein Binding to Small Molecule Inhibitors

### 4.1. The Impact of the Lid on the Binding of the Porphyrin Compound Nutlin-3a

**Figure 4:**
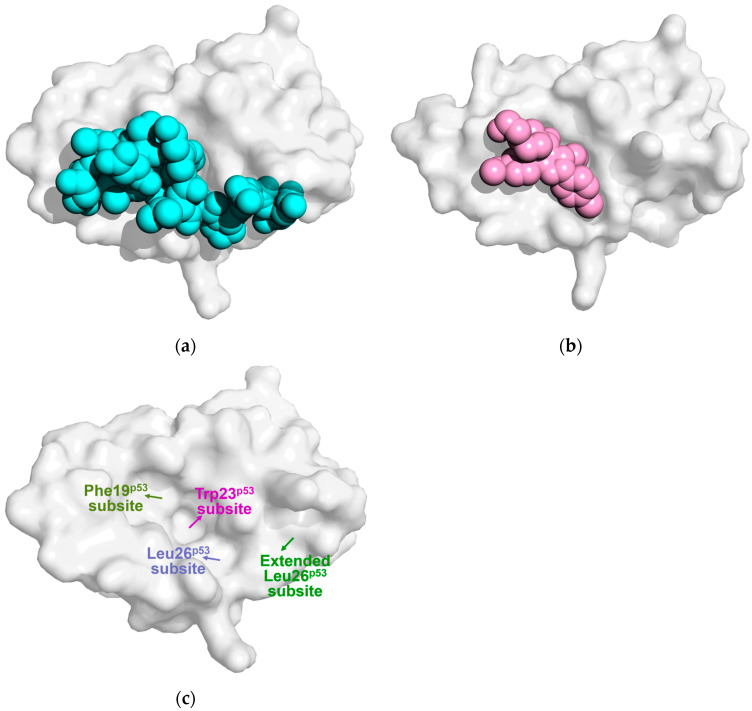
The surface of Mdm2^26–109^ forms corresponding binding pockets upon interaction with ligands. Binding of Mdm2^26–109^ with (**a**) p53 (cyan, PDB ID: 1YCR [17]) and (**b**) Nutlin-2 (pink, PDB ID: 1RV1 [50]). (**c**) The yellow, magenta, and light blue arrows indicate the positions of Mdm2’s three p53-binding subsites: Phe19^p53^, Trp23^p53^, and Leu26^p53^. The green region highlights the extended Leu26^p53^ subsite.

Nutlins are the first class of small molecule inhibitors that exhibit strong affinity for Mdm2 [50,51]. Nutlin-2 and Nutlin-3a, which are structurally similar, both display significant anti-tumor activity, with Nutlin-3a being the most widely used Mdm2 inhibitor in research. Both Mendoza-Martinez [35] and Showalter [22] have noted that, similar to apo-Mdm2, the lid of Mdm2 bound to Nutlin-3a exists in a dynamic equilibrium between “closed” and “open” states. Mendoza-Martinez’s ITC measurements [35] showed that the presence of the lid increases Mdm2’s affinity for Nutlin-3a by 1.2 to 2.3 times. Furthermore, MD simulations indicated that Nutlin-3a binds to the “open” state of Mdm2 with a 0.5 kcal/mol lower binding free energy compared to the “closed” state. As a result, the proportion of the “open” state increased from 7% in apo-Mdm2 to approximately 15%. These findings suggest that Mdm2 modulates its binding to Nutlin-3a through lid closure, with Nutlin-3a preferentially binding to the “open” state of Mdm2. Showalter et al. [22] compared the co-crystal structures of Mdm2/Nutlin-2 (PDB ID: 1RV1 [50]) and Mdm2/p53p (PDB ID: 1YCR [17]), as shown in Figure 4a,b. They found that Nutlin-2 occupies only 600 Å^3^ in the Mdm2 binding pocket, whereas p53p occupies a much larger volume of 1530 Å^3^. The p53p peptide extends from the Mdm2 binding pocket to the C-terminal region of the lid, enabling it to bind exclusively to the “open” state of the lid. In contrast, Nutlin-2 and Nutlin-3a are deeply embedded within the binding pocket, leaving space on the surface of Mdm2’s binding pocket to accommodate part of the lid. As a result, when Nutlin-3a binds to Mdm2, it does not induce the lid to transition to the “open” state, unlike p53. According to Schon et al. [52], the shorter p53p^17–26^ binds to Mdm2 with a lower binding enthalpy and a 10-fold stronger affinity than p53p^15–29^. Similarly, smaller molecules like Nutlin-3a and TEMPOL can bind to Mdm2 without causing the lid to open, thereby largely bypassing the lid’s role in ligand selectivity.

In the study by Bueren-Calabuig et al. [33], it was observed that the lid of Mdm2 bound to Nutlin-3a is generally more disordered compared to the lid of apo-Mdm2. Specifically, the lid region in direct contact with the ligand (residues 9–14) showed reduced flexibility, while the remaining regions of the lid displayed increased flexibility, resulting in an overall trend of increased flexibility in the lid. Their analysis further revealed the interaction pattern between the lid and Nutlin-3a: when Nutlin-3a binds to Mdm2, hydrogen bonds were observed between lid residues Asp11 and Glu23 with core residues of the Mdm2 binding pocket (Asp11 with His96 and Glu23 with Arg105), as well as between Thr10 and the imidazolinone ring of Nutlin-3a. Additionally, hydrophobic interactions were formed between lid residues Thr10, Val14, Ile19, Pro20, and Glu23 with the chlorophenyl ring of Nutlin-3a, collectively stabilizing the binding of Nutlin-3a to Mdm2.

Zhan et al. [26] used SPR technology to determine the K_d_ values of Nutlin-3a binding to Mdm2 and Mdm2^pS17^, both in complex with the lid, which were 83 nM and 108 nM, respectively. Phosphorylation of Ser17 slightly inhibited Mdm2’s binding to Nutlin-3a. However, Verkhivker [32], through MD simulations and molecular docking, found that in the Mdm2^pS17^ phosphorylation model, the lid adopts a stable “closed” conformation. In this state, the phosphorylated pSer17 forms a strong salt bridge with Lys94, while Pro20 on the lid occupies the Leu26^p53^ subsite, significantly hindering Nutlin-3a binding. In contrast, in the Mdm2^S17D^ simulated phosphorylation model, residues 16–20 move away from the binding pocket, causing Pro20 to no longer block the Leu26^p53^ subsite, as shown in Figure 4c. This “semi-closed” conformation opens the Mdm2 binding pocket partially, facilitating more effective binding with Nutlin-3a.

### 4.2. The Effect of the Lid on the Binding of Piperidone-Based Inhibitors

**Figure 5:**
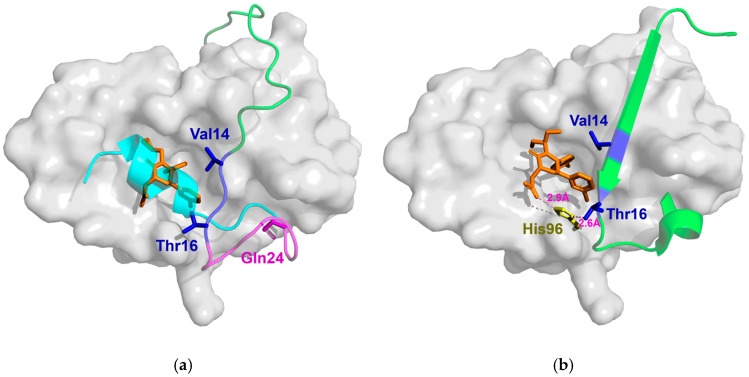
Crystal structures of Mdm2 in complex with Pip1 and Pip2. (**a**) The crystal structure of Mdm2^1–109^ bound to Pip1 (orange, PDB ID: 2LZG) and p53p^15–29^ (cyan, PDB ID: 1YCR). The Mdm2 lid is composed of an α-helix (magenta, residues 17–24), a short β-strand (dark blue, residues 14–16), and an unordered short chain (green, residues 1–13), partially occluding the Mdm2 binding pocket. (**b**) The crystal structure of Mdm2^6–111^ in complex with Pip2 (PDB ID: 4HBM). The green HT structure represents the Mdm2 lid (residues 6–24), while the orange Pip2 occupies the Mdm2 binding pocket. The para-chlorophenyl group of Pip2 is positioned between Val14 and Thr16 of the lid, and the carboxyl group of Pip2 forms a hydrogen bond with His96 of Mdm2 (pink dashed line).

Michelsen et al. [19] used X-ray crystallography, NMR, and biophysical experiments to demonstrate that the piperidone inhibitors Pip1 and Pip2 bind to Mdm2, inducing structural changes in the Mdm2 lid. Specifically, residues 14–16 of the lid form a short β-strand, while residues 17–24 adopt a short α-helix, partially occluding the Mdm2 binding pocket and hindering p53 binding, as shown in Figure 5a. Similar to Pip1, the para-chlorophenyl group of Pip2 is positioned between the side chains of Mdm2’s Val14 and Thr16, forming a hydrogen bond with the ε-nitrogen of His96, as shown in Figure 5b. Subsequent MD simulations conducted by Bueren-Calabuig et al. [33] revealed that Pip2 exhibits higher binding affinity for Mdm2 with an intact lid, whereas its affinity significantly decreases when the lid is partially or fully removed. This phenomenon primarily arises from the interaction between Pip2 and the lid region of Mdm2. Specifically, upon binding, the Glu23 residue in the lid forms a stable salt bridge with Arg97 at the binding site, while the Gln24 residue engages in hydrogen bonding with the backbone of Pro20, Ile19, and Thr16, thereby establishing a hydrogen bond network. These interactions stabilize the α-helix/β-turn (HT) motif formed by residues 14 to 24 of the lid. Moreover, hydrophobic interactions between the para-chlorophenyl group of Pip2 and both Val14 and Thr16 further reinforce the stability of the HT structure. When the lid is truncated at residue 17, Pip2 loses its ability to bind directly to the lid, underscoring the essential role of the complete lid structure in facilitating the interaction between Pip2 and Mdm2.

Additionally, Mendoza-Martinez et al. [35] found that the binding free energy of AM-7209, a piperidone derivative, to the “open” state of Mdm2 is 3 kcal/mol lower compared to its binding to the “closed” state. Binding of AM-7209 to Mdm2 also increases the proportion of the “open” conformation from 7% to approximately 90%, while Nutlin-3a binding only increases this to 15%. This suggests that, like Nutlin-3a, AM-7209 preferentially binds to Mdm2 in its “open” conformation. ITC titration results further reveal that truncation of the Mdm2 lid increases the K_d_ for AM-7209 binding by 250-fold, with a marked increase in binding enthalpy and a decrease in binding entropy. These findings suggest that AM-7209 enhances the rigidity of the lid region, reducing the binding entropy.

### 4.3. The Impact of the Lid on the Binding of Other Inhibitors

Bista et al. [53] developed the compound KK271 using a pharmacophore model based on key hydrophobic interactions in the p53/Mdm2 complex. KK271 is the first reported p53/Mdm2 inhibitor that directly interacts with the transiently folded α-helix in the lid region. The crystal structure of the Mdm2/KK271 complex shows that two KK271 molecules bind to Mdm2. One molecule occupies the Phe19^p53^, Trp23^p53^, and Leu26^p53^ subsites, causing the Tyr100 side chain to shift away from the Mdm2 binding pocket and resulting in an “expanded Leu26^p53^ subsite”. The second KK271 molecule fits into this expanded pocket through its benzyl group. Bista et al. synthesized YH300 by linking the benzyl group of the first molecule to the phenyl group of the second using a short methylene linker. YH300 demonstrated twice the binding affinity for Mdm2 compared to KK271. The crystal structure of the Mdm2/YH300 complex reveals that the expanded Leu26^p53^ subsite is occupied by a 4-fluorophenyl group. Additionally, the N-terminal lid residues 19–24 partially clash with the 4-fluorophenyl group, forming a concave pocket on the Mdm2 surface. Furthermore, Bueren-Calabuig et al. [33] performed MD simulations and found that when Mdm2 binds to the benzodiazepine compound 1,4-benzodiazepine-2,5-dione (Bzd), the flexibility of the lid region (residues 8–18) significantly decreases. In this state, the Val14 and Ile19 residues of the lid form hydrophobic interactions with the phenyl ring of Bzd, while the amino groups (-NH) on the Thr16 and Ser17 main chains form stable hydrogen bonds with the carboxyl group of Bzd. These interactions contribute to the stabilization of the Mdm2/Bzd complex.

In summary, the impact of the lid on different inhibitors varies. Most inhibitors influence the transition between the lid’s “closed” and “open” states, through mechanisms such as hydrophobic interactions between the lid residues and inhibitor groups, inhibitor-induced conformational rearrangements of the lid, or steric hindrance from certain lid regions when interacting with small molecules. Notably, residues 17–24 of the lid adopt different conformations when binding to various inhibitors, which may serve as a critical consideration in the design of Mdm2/MdmX-targeting inhibitors. In inhibitor design, while optimizing interactions with key residues in the binding pocket, it may be advantageous to focus on the interaction between the inhibitor group and the lid. By exploiting strong hydrophobic interactions to target the C-terminus of the lid and minimizing polar contacts with its hydrophilic groups, the inhibitor’s affinity could be significantly enhanced.

## 5. Conclusions and Perspectives

To date, research on the MdmX lid remains limited, whereas there is a relatively larger body of work on the structure and function of the Mdm2 lid. The impact of the lid on Mdm2 protein binding to ligands can be summarized as follows: (1) Both Mdm2 and MdmX lids exist in a dynamic equilibrium between “open” and “closed” conformations. The “open” state, which facilitates p53 binding, represents approximately 10% of the ensemble, while the “closed” state, which inhibits p53 binding, accounts for around 90% of the ensemble. (2) Some studies suggest that the lid regulates the size of the Mdm2 binding pocket by modulating the orientation of the Tyr100 side chain, thereby influencing Mdm2’s binding to p53 or other ligands. (3) Phosphorylation of Ser17 on the lid can shift the equilibrium between the “closed” and “open” states by interacting with residues within or near the Mdm2 pocket, such as Lys94 and His96, thereby modulating p53 binding. (4) However, other research proposes that lid phosphorylation does not directly affect the interaction between the lid and the Mdm2 binding pocket. (5) More recent studies have indicated that, although phosphorylation of the lid does not alter the Mdm2 binding pocket, it modulates the release of ubiquitin by UBCH5a through the Mdm2 RING domain, thereby enhancing p53 ubiquitination and subsequent degradation. The effect of the lid on Mdm2 binding with different inhibitors varies significantly: (6) For smaller ligands such as Nutlin-3a, these molecules can bind to Mdm2 without displacing the lid, interacting with lid residues through hydrophobic forces. As a result, the presence of the lid slightly enhances the binding affinity of these ligands to Mdm2; (7) Piperidone-based inhibitors, including Pip2 and AM7209, predominantly interact with lid residues, inducing structural rearrangements in residues 17–24 that lead to the formation of an α-helix/β-turn motif. This structural reorganization strengthens the interaction between the lid and the ligand, thereby enhancing the binding affinity of Mdm2 for these inhibitors.

Currently, there are only two studies focusing on the MdmX lid. The findings of Chen et al. contradict the prevailing views on the Mdm2 lid. In their study, truncation of the MdmX lid slightly reduced the binding affinity between MdmX and p53p, with minimal impact on the overall protein–p53 interaction. In contrast, Chan et al. highlighted that the MdmX lid itself has a limited effect on p53p binding. However, they found that MdmX can regulate the “closed” and “open” states of the lid through phosphorylation at two distinct sites within the binding pocket, Tyr99 and Tyr55, thus modulating its interaction with p53. Phosphorylation at Tyr99 shifts the lid toward the “closed” state, inhibiting MdmX-p53 binding. In contrast, phosphorylation at Tyr55 maintains the lid in the “open” state, facilitating MdmX-p53 binding and subsequently decreasing p53 activity.

The role of the Mdm2 lid in modulating the binding of p53 or Mdm2 inhibitors remains a topic of significant debate, with the underlying regulatory mechanisms still not fully understood. The primary source of discrepancy lies in the disordered nature of the lid, which continuously undergoes conformational changes. This inherent flexibility severely limits the selection of research methods, making MD simulations the dominant tool for exploring this issue. However, several factors, such as the timescale, constraints applied, and whether the system has reached equilibrium, critically influence the results of MD simulations. Even slight adjustments to any parameter can lead to substantial variations in outcomes, increasing the complexity and uncertainty of studying this phenomenon. Moreover, research on the MdmX lid is relatively limited, and no detailed studies have yet addressed how specific phosphorylation modifications to the lid affect MdmX’s binding to p53. This gap is primarily due to the presence of multiple potential phosphorylation sites within the MdmX lid (e.g., Thr2, Ser3, Ser5, Thr6, Ser7, Ser11, Thr12, Ser13, Ser15, Ser20), whose diversity complicates the study of lid phosphorylation. Beyond phosphorylation, the role of other post-translational modifications, such as acetylation, methylation, and glycosylation, in regulating the Mdm2 and MdmX lids remains largely unexplored. These modifications could offer deeper insights into their interactions with p53 and may represent novel therapeutic targets for cancer treatment. In conclusion, the exploration of the structure and function of the Mdm2/MdmX lid necessitates a thorough consideration of the intrinsic properties of the target protein, along with the investigation of various post-translational modifications. This approach should be integrated with structural and biochemical data derived from techniques such as NMR, X-ray crystallography, and ITC. During MD simulations, careful attention must be paid to the appropriate parameter settings, while in vivo and in vitro experimental validation should be conducted to ensure more comprehensive and reliable conclusions.

Current research indicates that the lid of Mdm2 and MdmX, along with the various modifications on the lid, plays a vital regulatory role in the interaction between Mdm2/MdmX proteins and p53. However, most existing Mdm2/MdmX inhibitors overlook this aspect, as they are typically designed and screened based on truncated, lidless Mdm2/MdmX models. Studies have shown that certain inhibitors can influence the transition between the “closed” and “open” states of the lid, thereby affecting Mdm2’s interaction with p53. Additionally, some inhibitors directly interact with lid residues, enhancing their affinity for the Mdm2 protein. These findings suggest that focusing on the lid region in inhibitor design could offer a promising therapeutic strategy. Specifically, by targeting the C-terminal end of the lid through strong hydrophobic interactions while minimizing polar contacts with its hydrophilic groups, this approach could facilitate the development of more potent MdmX inhibitors or dual Mdm2/MdmX inhibitors. Such strategies hold the potential to overcome the limitations of existing inhibitors and offer new avenues for cancer treatment.

In conclusion, a comprehensive study of the lid domains of Mdm2 and MdmX, along with the regulatory mechanisms of their various modifications on p53 binding, will provide crucial theoretical and experimental foundations for the development of novel cancer inhibitors. This research will not only enhance our understanding of the functional roles of Mdm2 and MdmX in relation to p53 but also open new possibilities for innovative approaches to cancer therapy.

## Figures and Tables

**Table 1:** SPR measurement of K_d_ values for Mdm2^1–109^ and Mdm2^25–109^ binding to different lengths of p53p.

	p53^19–26^ (nM)	p53^17–28^ (nM)	p53^15–29^ (nM)
Mdm2^1–109^	88,300 ± 3700	2250 ± 100	1260 ± 70
Mdm2^25–109^	39,600 ± 2000	404 ± 19	184 ± 14

## Data Availability

Not applicable.
